# SNOMED CT – advances in concept mapping, retrieval, and ontological foundations. Selected contributions to the Semantic Mining Conference on SNOMED CT (SMCS 2006)

**DOI:** 10.1186/1472-6947-8-S1-S1

**Published:** 2008-10-27

**Authors:** Stefan Schulz, Gunnar O Klein

**Affiliations:** 1 Institute of Medical Biometry and Medical Informatics, University Medical Center, Freiburg, Germany; 2 Department of Microbiology, Tumor, and Cell Biology, Karolinska Institutet, Sweden

## 

Medical science and health care both possess a strong tradition of structuring their terminological knowledge through controlled vocabularies, such as nomenclatures, thesauri, or classification systems [1-6]. The availability of such resources mirrors the need for sharing a common understanding of the employed terms and the semantic relations holding between them. Community-wide efforts such as the Unified Medical Language System (UMLS) [7], have reached a level of both coverage and depth today that is unmatched within many if not most other scientific disciplines.

The reason behind investing such a tremendous effort into those terminological systems is evident: They are intended to provide support for a wide range of different tasks, such as unambiguous, focused access to patient data for retrieval and decision support, the comparison of clinical cases, the retrieval of similar cases, clinical epidemiology and research, billing and accounting activities, the generation of health statistics from relevant and valid data, semantic interoperability between several (thematically overlapping) data sources, the quality management of medical services, as well as the structuring of scientific literature repositories and experimental databases.

So, the question may be raised whether the current biomedical terminological resources are already sufficient to properly serve the expressed needs. Basically, all terminology systems in current routine use rest on informal specifications. Their semantics are essentially rooted in a human understanding of natural language and implicit assumptions about the taxonomic, partonomic and other unspecified relations between terms. Interpreting such relations in light of a particular search, a decision support problem or, even more challenging, the drawing of ad hoc inferences often leads to strange and erroneous results. This is usually due to a lack of any rigid, formal semantics and ontological foundation underlying the respective terminological systems, an issue that has been increasingly addressed in recent years.

Whereas current biomedical terminology systems such as ICD, ICF, LOINC, CMPU, ICNP and many others are highly focused on quite well-delimited tasks and subdomains, the vision of a universal clinical terminology, covering a broad range of health-related domains and meeting the needs of all health professionals, has stimulated numerous health informatics research activities.

Is this vision now being materialized by the new health terminology SNOMED CT? [8]

During the last two decades, SNOMED (Systemized Nomenclature of Medicine) has been growing from a pathology-centered vocabulary to a comprehensive, structured clinical terminology. Even though SNOMED CT is still rooted in a strong legacy, it is increasingly subscribing to principles of logics and ontology. This fact, together with its impressive number of terms in most areas of medicine and health care has lead to a growing international interest. Nine countries have so far joined the International Health Terminology Standards Development Organization IHTSDO, a non-profit association founded in 2007 with the task of the development, quality assurance and distribution of SNOMED CT.

However, there are still only very few prototypical implementations of SNOMED CT in clinical settings, the feasibility of such a comprehensive terminology as basis for the whole health delivery process is still subject to discussion, and several shortcomings, regarding both SNOMED CT architeture and content, still persist [9].

The papers in this supplement of *BMC Medical Informatics and Decision Making *are extended and updated contributions to the Semantic Mining Conference on SNOMED CT (SMCS 2006), organized by the European Union Network of Excellence "Semantic Interoperability and Data Mining in Biomedicine" in October 2006.

It was the first European forum on SNOMED CT for health policy makers, clinicians, nurses, system developers, computer scientists, terminologists and translators. A number of prominent invited speakers provided overviews of the current efforts and developments in the context of SNOMED CT and many scientific contributions illuminated ongoing research on SNOMED CT. Out of the 22 scientific papers and posters published in the proceedings of this conference, eight were selected for this special issue by the program committee due to their scientific excellence. This selection mirrors the different research strands on SNOMED CT and represents a broad range of countries, viz., The Netherlands, USA, France, Australia, Switzerland, Sweden, UK, Hungary, and Germany. We want to thank the reviewers for their in-depth work in reviewing first the conference submissions and then again the selected contributions. The following contributions are included in this special issue:

In their article *Forty years of SNOMED: a literature review *[10] Ronald Cornet and Nicolette de Keizer provide an overview of published studies on SNOMED over a period of 40 years, reflected in scientific publications. They found that most studies concern SNOMED in theory and a minor number provides an account of the use of SNOMED in practice. (This is also a clear tendency regarding the papers in this special issue).

A major challenge of the adoption of a world-wide terminology is the use of legacy terminologies tailored and optimized to meet specific coding and documentation requirements. Therefore, a seamless migration from proprietary solutions to a common standard requires high-quality rules for manual cross-terminology mapping as described by Geraldine Wade and Trent Rosenbloom in their paper *Experiences Mapping a Legacy Interface Terminology to SNOMED CT *[11] which emphasizes the value of discoveries resulting from this mapping as important contributions to the refinement of SNOMED CT.

The relation between SNOMED CT and a legacy terminology is also addressed by Iulian Alecu, Cedric Bousquet, and Marie-Christine Jaulent in their paper *A case report: Using SNOMED CT for grouping Adverse Drug Reactions Terms *[12]. The authors provide evidence that the logical structure of SNOMED CT can be employed to improve term grouping and retrieval in the WHO Adverse Reaction Terminology, important for clinical trials and medical care.

Another mapping experience is reported by Yefeng Wang, Jon Patrick, Graeme Miller, and Julie O'Hallaran in *A Computational Linguistics Motivated Mapping of ICPC-2 PLUS to SNOMED CT *[13]. The authors compare different terminology mapping approaches including language engineering methods and also address the problem arising from the fact that one source concept maps to the coordination of two target concepts.

Computational linguistics approaches are also employed by Patrick Ruch, Julien Gobeill, Christian Lovis, and Antoine Geissbühler. [14] In their contribution *Automatic Medical Encoding with SNOMED Categories *they present two information retrieval approaches that address both the retrieval of SNOMED CT concepts and the automated encoding of free text and report on a first evaluation of a prototype.

The encoding of clinical data using information models and archetypes is addressed by Erik Sundvall, Rahil Qamar, Mikael Nyström, Mattias Forss, Håkan Petersson, Hans Åhlfeldt, and Alan Rector. In their paper *Integration of Tools for Binding Archetypes to SNOMED CT *[15] they present an approach that supports this task. They also discuss the yet unresolved problems of binding clinical information models to SNOMED CT and the control of post-coordination of concepts.

Two articles deal with the ontological background of SNOMED CT, an issue that has repeatedly been advocated in recent years. [9,16-18]

In the first one, entitled *Ontological analysis of SNOMED CT *[19], Gergely Héja, György Surján, and Péter Varga perform an analysis of the structure of SNOMED CT based on the formal top-level ontology DOLCE. They present a typology of errors occuring when the SNOMED CT hierarchies are submitted to formal ontological scrutiny and provide suggestions of how to avoid these errors.

In the second one, *Formal Representation of Complex SNOMED CT Expressions *[20], Stefan Schulz, Kornél Markó, and Boontawee Suntisrivaraporn focus on the representation of complex events and procedures, highlight the limited expressiveness of SNOMED CT regarding negations and formally describe the ambiguities in the representation of complex concepts.

Due to the recently facilitated access to the SNOMED CT sources, research activities are increasing all over the world and the interchange between the SNOMED CT community and the academic world is strengthening. Evidence for this is given by the recent MEDINFO, MIE and AMIA conferences as well as by the recent AMIA KR-MED 2008 conference on "Representing and Sharing Knowledge using SNOMED CT".

We hope that you will enjoy this special issue and that it helps you keeping track of some of the fascinating research in applied terminology, ontology and clinical terminologies.

## Competing interests

The authors declare that they have no competing interests.
